# ACT-R based human digital twin to enhance operators’ performance in process industries

**DOI:** 10.3389/fnhum.2023.1038060

**Published:** 2023-02-08

**Authors:** Bharatwaajan Balaji, Mohammed Aatif Shahab, Babji Srinivasan, Rajagopalan Srinivasan

**Affiliations:** ^1^Department of Chemical Engineering, Indian Institute of Technology Madras, Chennai, India; ^2^Department of Applied Mechanics, Indian Institute of Technology Madras, Chennai, India; ^3^American Express Lab for Data Analytics, Risk and Technology, Indian Institute of Technology Madras, Chennai, India

**Keywords:** operator performance, training, human digital twin, ACT-R, eye tracking, safety

## Abstract

To ensure safe and efficient operation, operators in process industries have to make timely decisions based on time-varying information. A holistic assessment of operators’ performance is, therefore, challenging. Current approaches to operator performance assessment are subjective and ignore operators’ cognitive behavior. In addition, these cannot be used to predict operators’ expected responses during novel situations that may arise during plant operations. The present study seeks to develop a human digital twin (HDT) that can simulate a control room operator’s behavior, even during various abnormal situations. The HDT has been developed using the ACT-R (Adaptive Control of Thought-Rational) cognitive architecture. It mimics a human operator as they monitor the process and intervene during abnormal situations. We conducted 426 trials to test the HDT’s ability to handle disturbance rejection tasks. In these simulations, we varied the reward and penalty parameters to provide feedback to the HDT. We validated the HDT using the eye gaze behavior of 10 human subjects who completed 110 similar disturbance rejection tasks as that of the HDT. The results indicate that the HDT exhibits similar gaze behaviors as the human subjects, even when dealing with abnormal situations. These indicate that the HDT’s cognitive capabilities are comparable to those of human operators. As possible applications, the proposed HDT can be used to generate a large database of human behavior during abnormalities which can then be used to spot and rectify flaws in novice operator’s mental models. Additionally, the HDT can also enhance operators’ decision-making during real-time operation.

## 1. Introduction

**FIGURE 1:**
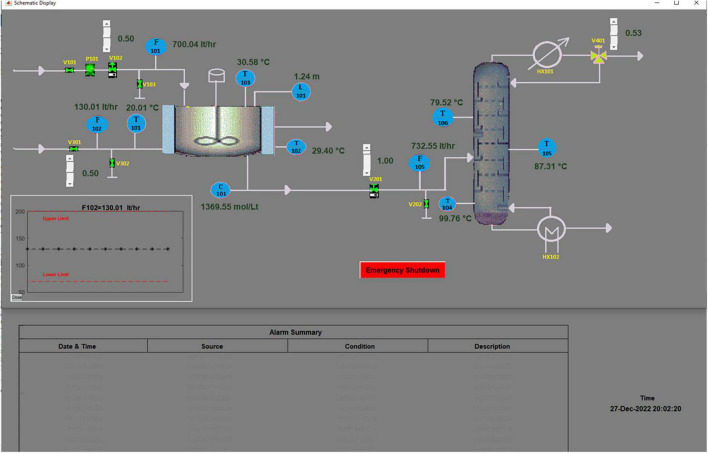
Human machine interface of the process simulator.

Modern industries utilize dependable equipment, cutting-edge automation and control strategies, and sophisticated safety management systems to ensure safety. Despite this, accidents continue to occur with varying degrees of severity (Khan et al., 2015). Studies indicate that human error accounts for most of these accidents—the root cause of 60–80% (Brauer, 2016). For instance, a study of severe industrial accidents involving hazardous chemicals from 2008 to 2018 revealed that over 76% of these can be traced back to human errors (Jung et al., 2020). Therefore, ensuring optimal human performance is key to ensuring safety in industries. Process industries rely on human-automation collaboration to ensure safety. The human operators in the control room monitor the process and intervene when abnormal situations arise (Shi et al., 2021). However, digitalization and the increase in sophistication of automation have altered the operator’s role and introduced additional challenges (Nazir et al., 2014; Di Flumeri et al., 2019). Operators need to gather, separate, and use information from several information sources to maintain optimal operating conditions (Zhang et al., 2020). Figure 1 shows an example of a human-machine interface that provides real-time information from a dynamically changing process. Using this, the operator has to continuously monitor the process, determine if it is normal, and intervene during abnormalities to ensure safety (as elaborated in section “2. Materials and methods”). This increases their cognitive workload, especially when they are not adequately trained or lack experience. Therefore, there is a critical need to characterize, understand, and improve operators’ performance, especially during abnormal situations.

There are two facets to addressing operators’ performance challenges—strengthening their competence and monitoring their behavior in the wild (Dai et al., 2016) in real-time. To enhance competence, researchers have primarily focused on providing suitable feedback to operators based on their performance. This requires performance evaluation in real-time. Approaches for evaluation typically rely on expert judgment (Nazir et al., 2015), which is subjective and ignores operators’ cognitive behaviors (Ikuma et al., 2014; Iqbal et al., 2018). Cognitive behavior is widely regarded as a substantial factor, especially during abnormal situations and accident causation (Zarei et al., 2021). There are hardly any studies that focus on real-time monitoring of operators’ performance, with the notable exception of Bhavsar et al. (2016) and Shi and Rothrock (2022). This is due to the lack of availability of operator behavioral information. Even if such data can be obtained, it is practically infeasible to train operators for every possible situation. As a result, it is necessary to develop tools with the ability to simulate operator behavior during a variety of industrial situations. Such a tool can forecast how an operator will perform during real-time plant operations (Kluge et al., 2014).

In this work, we develop a human digital twin that simulates control room operators’ behavior during abnormal situations. In recent years, researchers have proposed human digital twins to understand human behavior (Hafez, 2021) and enhance performance in various fields, including healthcare, sports (Miller and Spatz, 2022), driving (Wang et al., 2022), and aviation (Meyer et al., 2020). HDTs have been suggested as a means to monitor human performance in the real world, identify under-performance, and promote overall system enhancement (Miller and Spatz, 2022) through various interventions such as interface design.

The successful development of HDTs requires information about human perceptual, cognitive, and behavioral needs. Our previous studies have demonstrated that eye tracking can provide insights into human operators’ cognitive behavior (Srinivasan et al., 2019; Shahab et al., 2021a). Eye tracking entails tracking the movement of the eye and identifying the person’s point of gaze. It is based on the “eye-mind” hypothesis, which states that eye movement serves as a trace of the human’s dynamic attention allocation (Just and Carpenter, 1980; Rayner, 1998). The human eye is made up of various components that work together to permit visual processing and perception, such as the pupil and retina. The sensitivity of the retina varies, with the fovea having the greatest. Light is focused on the fovea by eye movements such as saccades, smooth pursuits, and fixations on visually interesting areas (referred to as areas of interest, AOIs). During a reading activity, for example, readers’ eyes concentrate on words and swiftly travel between them *via* saccades. The most common approach to eye tracking is video-based monitoring of eye movements (Holmqvist et al., 2011). Here a pattern of infrared light illuminates the eye. The reflected light is captured by a camera which produces the image of the eye. This image is analyzed using proprietary image analysis methods to estimate a gaze vector. A typical eye movement comprises of fixations and saccades (Holmqvist et al., 2011). Fixation is the interval during which the eye stays stationary and is required to process the information provided on the Human-Machine Interface (HMI). A saccade is a movement of the eye from one focus to another.

Our studies with control room operators reveal distinct mental models of expert and novice operators (Sharma et al., 2016; Shahab et al., 2021b). Such studies of mental models can reveal shortcomings in operators’ understanding of the process and can be used to enhance their skills (Shahab et al., 2022a). Based on the operator’s eye gaze patterns, we have developed quantitative metrics, such as the association metric, which quantifies the operator’s use of critical sources of information, and the salience metric, which evaluates the operator’s proactive monitoring strategy (Shahab et al., 2021c). Based on these previous experiences, in this work, we develop an HDT that seeks to replicate the human operator’s response during various process abnormalities. We validate the developed HDT using the eye gaze behavior of real human operators.

The developed HDT can address the challenges associated with operator performance during training as well as during real-time plant operation. Firstly, during training, the developed HDT can simulate the human operators’ control actions and eye gaze behavior at different levels of expertise. This simulated behavioral data may then be utilized to create a vast knowledge base which can then provide the basis to spot flaws in the mental models of novice operators as they deal with abnormal conditions. Secondly, the HDT can be used to predict the behavior of the human counterpart during real-time plant operation. When an abnormal situation occurs, the HDT can be used to predict whether the human counterpart can deal with the situation. In addition, it can also provide relevant cues to the human operator, thereby assisting in enhancing their situational awareness. Adequate situational awareness and skills are crucial, especially when dealing with abnormal situations (Salmon et al., 2006; Srinivasan et al., 2022).

## 2. Materials and methods

In this section, we discuss the development and validation of the proposed human digital twin for process control applications. A human digital twin (HDT) is the digital representation of a real-world human (Miller and Spatz, 2022). An HDT with cognitive skills and human-like intelligence can tackle complex problems. Such HDTs can be created using cognitive architectures. Cognitive architectures may be conceived of as a model for human behavior simulation. The cognitive architecture consists of modules for perception and action to imitate overall human behavior during a task. In this work, we used the ACT-R (Adaptive Control of Though-Rational) cognitive architecture to develop the human digital twin.

### 2.1. ACT-R

ACT-R is a popular cognitive architecture that is based on a rigorous theory of human cognition (Anderson, 2007). It explains human cognition by a model of the knowledge structures that underlie cognition (Anderson et al., 2004; Anderson, 2007). ACT-R consists of a symbolic layer and a sub-symbolic layer which are tightly coupled together. The ACT-R’s symbolic layer is based on a modular representation of how the human brain processes information (Albrecht and Westphal, 2014). There is a visual module for perceiving visual cues, a motor module for controlling actions, and a goal module for keeping track of desired outcomes. The symbolic layer stores these modules and also the details of the underlying memory structure. There are two types of memory structures in ACT-R: declarative memory and procedural memory. The factual information maintained in human memory is known as declarative memory. In ACT-R, it is the standard system for storing and retrieving information (Fleetwood and Byrne, 2006). It aids in the definition of things, events, and processes, as well as their traits and inter-relationships. Declarative information is represented in structures called chunks. A chunk is defined by its type and its slots. Chunk types can be regarded as categories (e.g., human beings), and slots as attributes of the categories (e.g., age or gender).

The procedural memory in ACT-R stores the procedures and skills necessary to achieve a given goal. These are stored as production rules, i.e., IF–THEN condition–action mappings that “fire” when the conditions are satisfied and execute the specified actions. The production conditions (the “IF” side of the production rules) are matched to a set of buffers that define the current state of the various ACT-R modules (such as the visual module). The buffers provide the interface between the production rules and ACT-R modules. Any production rule whose conditions are matched is fired, which may alter the contents of buffers by instructing modules to do a job, such as shifting the user’s attention or retrieving a chunk of declarative memory.

**FIGURE 2:**
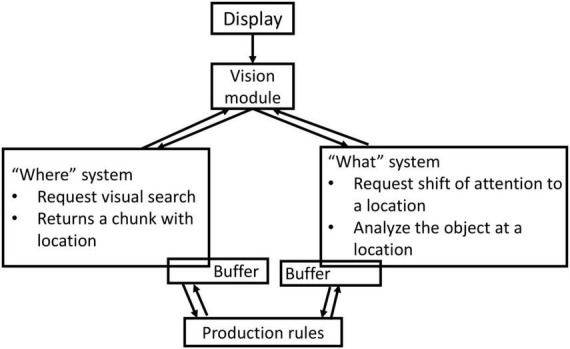
Working of ACT-R’s vision module.

Given the visual nature of the graphical user interfaces, the user’s attention to the display is of key importance for the HDT of a control room operator. The vision module takes care of what ACT-R sees on the display. As shown in Figure 2, it consists of two systems: the “where” system and the “what” system (Anderson et al., 2004). When a production makes a request to shift attention, the “where” system performs a visual search and returns a chunk representing the location of the object on the display (Figure 2). The “where” system helps the ACT-R with the knowledge of the location of various objects on the display. To identify objects, a request to the “what” system is made. When a chunk representing a visual place is given to the “what” system, the “what” system will move the attention to that position, analyze the object that is there, and create a declarative memory chunk that represents the object (Anderson et al., 2004), as depicted in Figure 2. The firing of production rules directs the “what” system to move attention from one location on the display to another. In this way, the vision module simulates fixations and saccades.

The symbolic layer of the ACT-R (declarative and procedural) is controlled by the sub-symbolic layer, whose parameters have to be optimized to the environment’s structure (Best et al., 2002). These variables represent the fundamental adaptivity, robustness, and stochasticity of human cognition, especially in settings that are characterized by unpredictable, chaotic, and incomplete information. These parameters control many production rules, and the important ones (related to current work) are listed in Table 1. For instance, the speed with which ACT-R performs motor activities is governed by burst-time and feature-burst-time, which decide the relaxation time after a motor activity. Similarly, the initiation-time parameter controls the preparation time for motor activity. Further, the retrieval of a chunk from the declarative memory depends on the retrieval threshold (τ). A chunk will only be retrieved if its activity is above τ. The activity of a chunk reflects the probability that a chunk will be helpful at a given time. The parameter :*l*f determines the speed of a chunk retrieval. The majority of learning processes in ACT-R are driven by sub-symbolic mechanisms (Best et al., 2002). In addition, they can be affected by the reward/penalty system, which provides feedback after the execution of production rules. The reward/penalty value (*R*) decides the likelihood that a production rule will be used in the future (Dimov et al., 2020).

**TABLE 1:** ACT-R parameters tuned for HDT of process operator.

S. No	ACT-R parameters	Value
1	Motor-burst-time	0.02
2	Motor-feature-burst-time	0.02
3	Motor-initiation-time	0.02
4	τ (retrieval threshold)	−0.5
5	*:lf* (determines speed of chunk retrieval)	1.15
6	Reward/penalty (*R*)	Varied
7	*K* (domain-specific parameters)	0.15
8	α (domain-specific parameters)	0.10

The ACT-R theory is embodied in software that is implemented in ANSI Common Lisp (Bothell, 2020). An ACT-R model needs to be customized for domain-specific applications. Next, we describe the model’s customization for process control tasks.

### 2.2. Human digital twin development

We have developed an HDT of a control room operator responsible for supervising a process plant. The chemical process consists of a Continuous Stirred Tank Reactor (CSTR) to react ethene and water and a distillation column for product separation and has been implemented in a process simulator developed in-house using MATLAB. A total of eleven process variables govern the process. The role of the operator is to monitor the process and take corrective actions during abnormal situations. The operator would use the HMI shown in Figure 1 for this purpose. The HMI mimics a distributed control system in a typical industrial context. Any process abnormality is notified to the operator by aural and visual alarms—a beep sound and a change in the color of the alarmed variable’s tag from black to red. The HMI also displays a summary of the alarms and their state (high or low), as seen at the bottom of Figure 1. When any abnormality occurs, the operator is required to intervene appropriately and control the process using the sliders in the HMI, which trigger the manipulation of the control valves in the process. The operator can also obtain time-based information on process variables and predict the effect of any action(s) they take using the process trend panel, as shown in Figure 1. Any process disruption must be managed within 2 min of its occurrence; otherwise, the process will automatically undergo an emergency shutdown. We next discuss the operation of the HDT.

**FIGURE 3:**
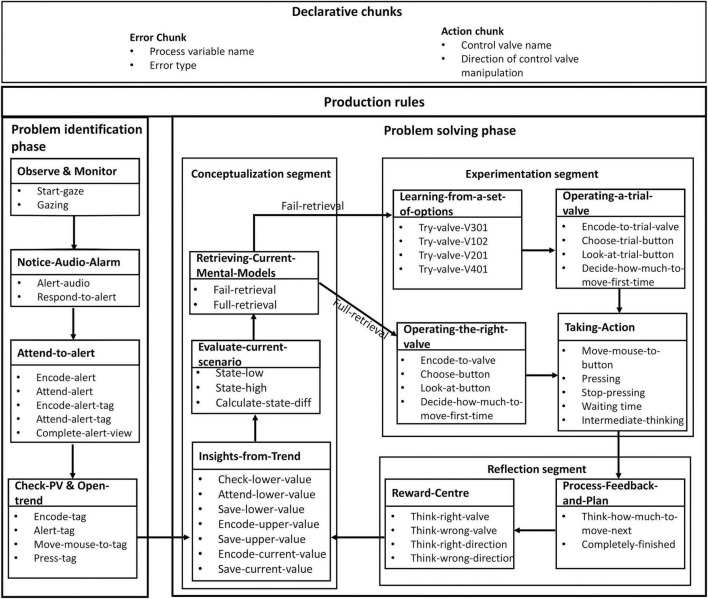
Production rules in the human digital twin of process operators.

Mimicking the human operator, the digital twin’s role is to monitor the process and intervene when an abnormal situation arises. For this purpose, the memory structures—declarative chunks and production rules—in the ACT-R’s symbolic layer are customized, as shown in Figure 3. The HDT has two declarative chunks—an error chunk and an action chunk—each having two slots. The slots of the error chunk store the name of the process variable that has an error (i.e., deviation) and the nature of the error (above or below the safe operation range). The action chunk stores the name of the control valve that has been operated and the direction of the move. The behavior of the process in response to the action is stored in the declarative memory. Thus, error chunks add different errors that arise in the process to the declarative memory of the model, while the action chunk enumerates all the actions the model has taken.

The production rules of the HDT help the digital twin deal with process abnormalities. Figure 3 shows the various production rules and indicates their goals (e.g., Observe and Monitor; Notice-Audio-Alarm; Attend-to-Alert). The production rules achieve their goal through various steps (e.g., Start-gaze and Gazing); further, the arrows indicate the flow of control between the goals. The HDT uses a total of 48 production rules to accomplish different goals during process abnormalities.

The HDT’s behavior during process abnormalities can be explained in two phases: Problem identification and Problem-solving. In the problem identification phase, the digital twin familiarizes itself with the abnormality in the process. It requires locating the source of disturbance and its deviation (high or low) by attending to the alarm panel and/or tags. The problem-identification phase has four goals and 13 production rules which are fired during process abnormalities (Figure 3). Once the digital twin familiarizes itself with the nature of the abnormality, it moves to the problem-solving phase (Figure 3). This phase can be described as a continuous cycle of Conceptualization, Experimentation, and Reflection (Li and Maani, 2011). Each of these phases has its own production rules, as shown in Figure 3. The problem-solving phase is cognitively more complex than the problem-identification phase.

For example, during normal process operation, the production rules in the “Observe & Monitor” goal direct the HDT to observe the process parameters (Figure 3). When an abnormal situation occurs, the HDT first familiarizes itself with the abnormality by firing production rules from the “Notice-Audio-Alarm” goal and “Attend-to-alert” goal in the problem identification phase. Then it attends to the alarm panel of the HMI to identify the source of disturbance by firing rules from the “Attend-to-alarm” goal. Next, the HDT fires productions from the “Check-PV & Open-Trend” goal to identify the tag corresponding to the process variable mentioned in the alarm and opens the trend of the process variable on the HMI.

The digital twin then proceeds to the conceptualization segment of the problem-solving phase. Conceptualization involves the understanding of the disturbance (grasping the root cause and magnitude of disturbance) and the available control actions which would return the process to the normal limits. It has three goals and a total of 12 production rules. For example, the firing of production rules from the “Insights-from-Trend” goal scans the trend panel for relevant information like the process variable’s current value and its normal operating conditions. The productions in the “Evaluate-Current-Scenario” goal help the HDT understand the nature (below or above the safe operating condition) and magnitude of the disturbance. With the knowledge of such finer details of the abnormality, the HDT starts proposing plans to control the disturbance by firing productions from the “Retrieving-Current-Mental-Models” goal (Figure 3). Here, the digital twin recalls or uses the mental models of the process currently in its declarative memory to solve the current disturbance (error chunk). If a useful chunk is available in the mental models (from the declarative memory), the “full-retrieval” production is called; else, the “fail-retrieval” production is called.

When the conceptualization segment has created a firm set of conclusions and actions, the HDT moves to the experimentation segment. This entails executing actions based on the decisions made in the conceptualization segment. The experimentation segment consists of four goals (1) Learning-from-a-set-of-actions, (2) Operating-a-Trial-Valve, (3) Operating-the-Right-Valve, and (4) Taking-Action. The firing of productions from the goals “Try-a-valve” and “Operating-the-Right-Valve” (Figure 3) depends on the declarative memory of the HDT. The digital twin can take action by firing any one of these productions. Once a valve is chosen, it is first manipulated slightly to get a sense of the calibration of the valve. By firing productions from the “Taking-Action” goal, the HDT moves the mouse to the desired button and manipulates the control valve by an appropriate amount. To see the effect of the valve manipulation, the human digital twin also waits for a short time. The HDT stores the information of the control valve operated and the resulting changes in the process variable in the working memory. If the HDT had fired the “full-retrieval” production in the conceptualization segment, then the productions from the goals “Try-a-valve” and “Operating-the-Right-Valve” would be fired sequentially in the experimentation segment. This signifies that the HDT has found a chunk related to the current disturbance in its declarative memory.

During the reflection phase, the results of the experimental phase are considered. If the desired outcomes are achieved, the initial perception and judgments are reinforced in the declarative memory. For instance, if the HDT successfully controls the process using a control action, then that control action will be stored in the declarative memory along with the disturbance, and a reward will be given to the HDT. In case the control action of the HDT is incorrect (i.e., fails to bring the variable within the normal limits), the HDT will receive a penalty for using that action. In the future, when the HDT is confronted with a similar abnormal situation, it will use the corrective action based on the reward/penalty it received in the previous such situations. In this way, the human digital twin establishes causal linkages between the control actions and their effect on the process and stores them as chunks in the declarative memory. The production rules of the “Reward-Centre” goal take care of the rewards/penalties (Figure 3). Based on the reward/penalty allotted by the “Reward-Centre,” the HDT iterates the Conceptualization-Experimentation-Reflection loop till the process reaches the normal state.

The production rules operate in tandem with the ACT-visual R’s module. As discussed previously, the visual module consists of two buffers—one for “where” and the other for “what” information. During process abnormalities, the firing of the production rule from the “Attend-to-alert” goal (Figure 3) triggers a shift of the HDT’s attention to the alarm panel. The production rule “Encode-alert” creates a visual chunk in the “where” buffer of the vision module with the information on the alarm panel location. Then, by firing the “Attend-alert” production rule, the HDT fixates on the alarm panel to obtain information about the disturbance. The firing of the “Encode-alert-tag” rule changes the information of the “where” buffer from the location of the alarm panel to the location of the alarmed process variable. The HDT moves its attention to the variable tag from the alarm panel by firing the “Attend-alert-tag” rule. In this way, the HDT generates fixations and saccades while dealing with process abnormalities.

As discussed previously, the symbolic layer of the ACT-R is tightly coupled with the sub-symbolic layer. To mimic the human operator’s behavior, it is important to include parameter values specific to process control tasks in the ACT-R model. We tuned some of the sub-symbolic parameters of the ACT-R according to the need in process control tasks, as shown in Table 1. The values for these parameters were chosen not to strictly replicate the performance of any one single participant (which could overfit the model) but to reflect typical responses. For instance, we tuned the parameters that account for the speed of motor actions (mouse clicks on the valves and process variable tags) of the ACT-R (Table 1), such as burst time, feature burst time, and initiation time. These parameters were given values lower than the default ACT-R value due to the simplicity of motor actions in the process control task. Similarly, the value of the parameter *:lf* is set to be slightly higher than 1 to mimic the difficulty faced by human operators in recalling relevant information during abnormalities.

In addition to the sub-symbolic parameters, the HDT also contains parameters that account for variability in the manipulation of the control valve by different operators, i.e., gentle or abrupt manipulation. When *n* is the total number of steps required to control a disturbance, the variability in human actions can be expressed mathematically as:


(1) $$s_{n}=1+k\times \frac{y_{d}^{n}}{1+y_{d}^{n}-y_{d}^{n-1}}\times s_{n-1}$$

(2) $$\epsilon =\alpha \times \left(y_{u}-y_{l}\right)$$

where *s*_*n*_ and *s*_*n*−1_ are the amount of control valve manipulation required at *n^th^* step and (*n* − 1)^*th*^ step, respectively. $y_{d}^{n}$ and $y_{d}^{n-1}$ are the deviation of process variable before the *n^th^* step and (*n* − 1)^*th*^ step, and *y*_*u*_ and *y*_*l*_ are the upper and lower limits beyond which the process variable is in alarm status, respectively. The amount of slider manipulation is modeled as a proportional controller with proportional constant *k*, where *k* defines the aggressiveness (in terms of the amount manipulated at each action) of the HDT while manipulating the control valve. A high value of *k* would imply that the digital twin manipulates the valve by a larger amount at each step while taking the control action and thus would make fewer steps to control the scenario. The parameter α measures the safety tolerance for a process variable in the HDT. For example, some operators would stop control actions when the process variable just reaches within the bounds of the normal operating limit, while others try to bring the variable to the midpoint of the normal range (i.e., the steady-state value). Thus, this perception of safety tolerance of the operator is captured through α as a percentage of the range of safe operating conditions. A large value of α would mean that the digital twin would move the process variable well into the safe operating range. Nominal values for *k* and α are shown in Table 1. In addition to these, ACT-R uses a reward/penalty center to improve its performance. The HDT receives a reward (*R*) for taking the correct control action and successful completion of the task, while it obtains a penalty for manipulating incorrect control action and automatic shutdown of the plant. Based on the feedback from the reward and the penalty center, the HDT updates its declarative memory and uses it to control the disturbance in the future.

Next, we discuss the methodology to validate the developed human digital twin using human subject studies.

### 2.3. Human digital twin validation

The aim of the HDT is to mimic the behavior of a human operator. So, the successful development of an HDT requires that the cognitive abilities must be comparable to those of the human operator. In this section, we discuss the validation of the proposed human digital twin. First, we generated the simulated operator behavior from the HDT. To compare cognitive capabilities, we compared the gaze of the HDT with the eye gaze behavior of human subjects who performed similar tasks.

### 2.4. Human digital twin simulations

**FIGURE 4:**
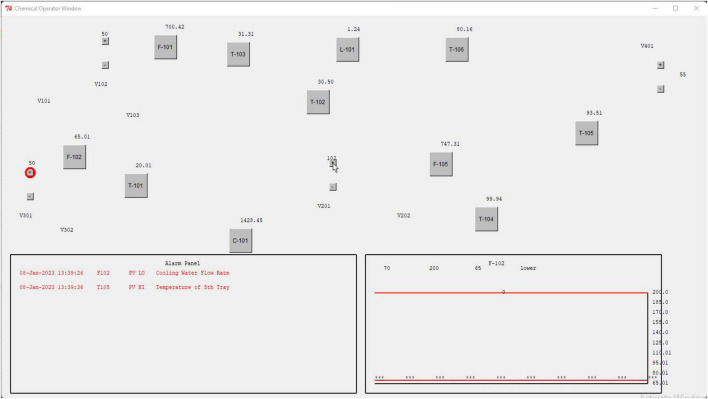
Human machine interface of the process simulator used by the HDT.

The chemical process simulation was implemented on MATLAB^®^ R2021b, while the ACT-R model was hosted by a Python^®^ script that also supported the HMI. Data was transferred between the MATLAB^®^ simulation and Python through a TCP/IP connection. Figure 4 shows the HMI created for the HDT model which is similar to that of the HMI used by operators. The human digital twin model was simulated for 76 runs, each with different values of rewards and penalties. During each run, the model learns to control the disturbance after a few trials. Here, a trial is the repetition of the same task by the model during a run. In total, this resulted in 426 different trials. Out of these, the HDT successfully completed the task in 350 trials and failed in 74. We ran the ACT-R simulation for a combination of three different values of the reward/penalty, i.e., 0.5, 5, and 10. For example, during a typical run, if the HDT takes a correct control action to control a disturbance, it receives a reward value of 0.5. For an incorrect control action, it obtains a penalty of –5. Similarly, for successful completion of the task, it gets a reward value of 5, and for automatic shutdown of the plant, it obtains a penalty of –10. If the feedback given to the HDT in the form of reward/penalty is not appropriate, the HDT may take many trials during a run to succeed in controlling the disturbance. In essence, the HDT’s reflection on its performance is impacted by modifying the magnitude of reward/penalty. This is in line with prior studies where it was reported that feedback is an important aspect of improving the operator’s performance during training (Kluge et al., 2009).

We recorded process variables data from the MATLAB^®^ process simulator, HDT actions in response to abnormalities, and HDT gaze data from the vision module of the ACT-R, as discussed in the preceding section.

### 2.5. Human subjects

We used data from the human subject study reported in our earlier work (Kodappully et al., 2016) to validate the HDT model. A total of 11 participants with a background in control systems were involved in the study. The participants performed similar disturbance rejection tasks as performed by the HDT—monitor the process and intervene during abnormalities. Prior to conducting tests, participants were given handouts that explained their responsibilities as operators, the technical details of the process, and how to interact with the HMI. In addition, a video tutorial was provided, which demonstrates the use of HMI to control the process. Task-specific instructions are shown on the screen prior to starting each task. These instructions include information about the nature of the abnormality, disturbed variable (s), and the control action to be used to bring the process back to normal. For instance, a typical task involves a disturbance in the cooling water flowrate, which results in a low or high alarm on the F102 tag that measures the cooling water flowrate. The participant is expected to monitor the process and execute corrective actions (manipulate control valve V301) using the HMI to bring the process back to a normal operating limit. The reader is referred to Kodappully et al. (2016) for details of the protocol followed. Overall, the participants performed 110 tasks involving various scenarios. During each task, we collected process data, operator actions, and eye tracking data (using the Tobii TX 300 eye tracker).

### 2.6. Data analysis

It is expected that the HDT should show similar cognitive behavior as its human counterpart. The eye gaze pattern serves as a trace of attention allocation by the human. Therefore, we compared the performance of the HDT and the human operator using eye gaze data. For analyzing the gaze data, we divided the HMI into various areas of interest (AOIs), such as process variable tags, control sliders, trend pane, and alarm summary. We term the process variables that are directly affected by a disturbance as the primary variables for that scenario and the rest as secondary variables (Kodappully et al., 2016). Similarly, tags and sliders are also categorized into primary tags, primary sliders (correct control action to bring the plant to the normal operating state), secondary tags, and secondary sliders. For instance, consider a task during which a disturbance occurs in the form of a decrease in coolant flow to the CSTR (F102), which causes low alarm F102. The corrective action is to increase the opening of the coolant flow valve (V301). During this task, the primary tag is the coolant flow tag (F102), the primary slider is the coolant flow valve (V301), and the primary trend is the trend of F102.

To compare the performance of the HDT and the human operator, we consider sequences containing two AOIs visited consecutively—we term these as a *2-tuple transition*. Similarly, three AOIs visited in succession are termed a *3-tuple transition*. We consider such transitions during various phases of a task, such as after the occurrence of the alarm till the first control action (diagnosis phase) and from the first action till the last action (execution phase). Prior studies have shown that operators use time-based information to foresee the effect of their control action (Yin et al., 2008; Shahab et al., 2022b). Therefore, we identified the percentage of gaze transitions involving the trend panel and primary variables for both the human operator and the HDT. These include two tuple transitions such as “trend panel→ primary slider,” “primary slider→ trend panel,” and “primary tag→ trend panel.” In addition, we also calculated three tuple transitions “trend panel→ primary slider→ trend panel” and “primary slider→ trend panel→ primary slider” during two different phases of the task as discussed above. For instance, the percentage of the two tuple gaze transitions “primary slider→ trend panel” in a particular phase (diagnosis or execution) is obtained by dividing the number of transitions from the primary slider to the trend panel by the total number of two tuple transitions that occurred during that phase. The gaze transitions between primary variables and the trend panel indicate that operators attend AOIs that are directly related to the disturbance, and they use a proactive monitoring strategy using the trend of the process variables. Such gaze behavior is commonly observed in expert operators. The expert gaze behavior is distinct from those observed in novice operators (Sharma et al., 2016). Next, we compare the results from the human operator and the HDT.

## 3. Results

In the following, the results obtained from the data gathered through the methods described above are analyzed.

### 3.1. Human operator behavior

Eleven participants played the role of control room operator in disturbance rejection tasks. Typically, at the beginning of the experiment, the participant, after going through the training phase and reading the task instructions, scans the schematic and identifies the tags and sliders mentioned in the instructions before starting the task. During the task, due to the disturbance, one or more process variables can go beyond normal operating limits, which would lead to alarm(s). The task of the participant is to bring the process back to normal by accurately diagnosing the cause of the abnormality and initiating necessary control actions using the sliders on the HMI. Once a disturbance occurred, participants typically used the trend panel to obtain information about the primary variables and then executed corrective actions using the primary slider to bring the process back to normal.

**FIGURE 5:**
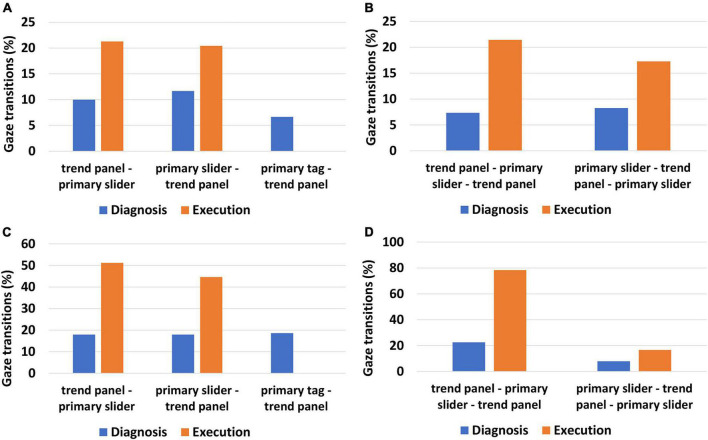
Gaze transition analysis during diagnosis and execution phase **(A)** two tuple gaze transition for human participants for successful trials **(B)** three tuple gaze transition for human participants for successful trials **(C)** two tuple gaze transition for the human digital twin for successful trials **(D)** three tuple gaze transition for the human digital twin for successful trials.

As discussed earlier, we calculated two tuple and three tuple transitions in two phases of the task—the diagnosis phase and the execution phase. The percentage of gaze transitions between the primary variables and the trend panel for all the human participants who successfully completed the task is shown in Figure 5. As depicted in Figure 5A, the two tuple transitions “trend panel→ primary slider” and “primary slider→ trend panel” is higher during execution than in the diagnosis phase. Similarly, as reflected in Figure 5B, three tuple transitions involving primary variables and the trend panel are higher in the execution phase. This is because, during diagnosis, participants identify the root cause of the disturbance and hypothesize about the correct action to control the disturbance. Accordingly, they locate the primary variables (such as the primary tag and slider) that help them control the disturbance. Further, as shown in Figure 5A, they also use the trend panel to obtain details of the deviation from the alarm limits, as depicted by the transitions from the primary tag to the trend panel during this phase. However, when operators execute the control action in the execution phase, the use of the trend panel becomes more crucial as it provides more information than any other AOIs (Kodappully et al., 2016). The trend panel contains information about the magnitude, status (high or low), and rate of change of the disturbed variable in response to the operator’s actions (use of sliders). As a result, during this phase, we see more transitions to the trend panel (Figures 5A, B), and the primary tag is no longer relevant as the disturbance has already been identified in the diagnosis phase. The same is also reflected by the absence of two tuple transitions “primary tag→ trend panel” in the execution phase (Figure 5A). Next, we discuss the gaze behavior of the HDT. It is expected that the HDT should follow the same gaze behavior as the human participants.

### 3.2. Human digital twin behavior

To compare the performance of the HDT with that of the human operators, we calculated the two-tuple and three-tuple transitions from the HDT gaze behavior during successful tasks. We obtained the gaze behavior of the HDT from the vision module of the ACT-R. The gaze transitions of HDT during success are shown in Figures 5C, D.

From Figure 5C, it can be observed that the gaze transitions from the “trend panel→ primary slider” are higher in the execution phase than in the diagnosis phase. Similarly, two tuple transitions “primary slider→ trend panel” are higher in the execution phase as compared to the diagnosis phase. This signifies that the HDT obtains most of the information from the trend panel during the execution phase. As discussed in the previous section, we observed the same pattern for human participants (Figure 5A). Also, there is a presence of gaze transitions from the primary tag to the trend panel in the diagnostic phase only (Figure 5C), which is in line with the behavior of human participants (Figure 5A). This is because the HDT focused only on the primary slider and trend panel in the execution phase highlighting the role of trend as a critical information source. Similar gaze behavior of the HDT can be observed for three tuple transitions (Figure 5D). The three tuple transitions “trend panel→ primary slider→ trend panel” is higher in the execution phase than in the diagnosis phase (Figure 5D), akin to the pattern observed for human participants (Figure 5B). The results indicate that the developed HDT mimics the behavior of its human counterparts. It may be noted that there is a difference in the magnitude of gaze transitions between human operators and the HDT (Figure 5). This could be due to the innate nature of human operators; for instance, even after completing a task, their eye gaze would continue scanning (random gaze behavior) rather than remain fixated on a specific AOI. The HDT does not model such behaviors.

**FIGURE 6:**
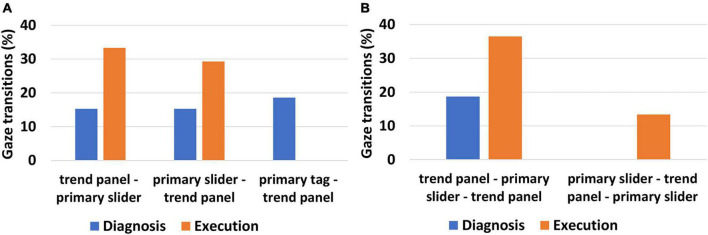
Gaze transition analysis of the human digital twin during diagnosis and execution phase **(A)** two tuple gaze transition for the HDT during failure trials **(B)** three tuple gaze transition for the HDT during failure trials.

In addition to the analysis of the trials where the human digital twin successfully completed the task, we also calculated HDT behavior during failure tasks (that resulted in automatic shutdown). The HDT failed in a total of 76 trials. We calculated the gaze transitions for these trials, which are shown in Figure 6. It is interesting to observe that even when the HDT failed in the task, the gaze resembles that of the human participants who successfully completed the task. The two tuple transitions “primary slider→ trend panel” is higher in the execution phase than in the diagnosis phase (Figure 6A). The same is observed for three tuple transitions trend “panel→ primary slider→ trend panel” which is higher in the execution phase (Figure 6B). This clearly suggests that the HDT attempted to orient the gaze pattern in accordance with the task but was unable to do so due to a lack of pertinent information in the declarative memory. Although the gaze transitions exhibit the same tendency for failure, there is a distinction when compared to HDT’s successful trials. By comparing Figures 5C, D, Figures 6A, B, it can be observed that the percentage of both two-tuple and three-tuple gaze transitions from primary variables to trend panel is higher during successful tasks than in failure tasks. The HDT learns to control the abnormality by offering penalties for incorrect actions and rewarding correct actions. Therefore, with learning, the HDT orients the gaze pattern and has higher gaze transitions between primary variables and the trend panel.

## 4. Discussion

In this work, we developed a human digital twin using the ACT-R cognitive architecture to simulate a control room operator who has to tackle process abnormalities. To our knowledge, this is the first reported research of its kind in which a human digital twin is developed to address human performance issues in the domain of process industries. The developed HDT has been validated using the human operator’s eye gaze. Our results indicate that the HDT’s behavior accurately replicates that of the human operators’. Specifically, gaze transitions between primary variables were found to follow the same patterns. For instance, in human operators, the gaze transitions between the trend panel and primary variables during the execution phase were higher than during the diagnosis phase (Figure 5A). This signifies that operators use the trend panel extensively when the requirement shifts from diagnosis to execution. Observing the trend panel helps in obtaining time-based information on the process variables. The same behavior is also observed in the HDT (Figure 5C). This indicates that the production rules defined in the ACT-R model to solve process abnormalities accurately represent the cognitive functions of the human operator. It is important to note that the HDT simulations were carried out by tuning only those parameters that give the HDT feedback to enhance its performance across trials. The parameters of the ACT-R vision module were unchanged, yet the model exhibits gaze patterns comparable to those of human operators. The HDT learns to control the process based on the rewards/penalty received, similar to how human operators improve their performance after obtaining appropriate feedback. These observations are also supported by the ACT-R model validation in other safety-critical areas such as driving and aviation (Salvucci, 2006; Park et al., 2018). For instance, the ACT-R driver model demonstrates the same gaze behavior as human drivers, i.e., mostly attending to the front of the vehicle and transitioning to other areas for better situational awareness. The ACT-R model also correctly predicted the gaze time on distant objects as observed for human drivers (Salvucci, 2006).

As an application, the developed HDT can be used to build an extensive (synthetic) knowledge base of operator behavior during various abnormal situations. Such a knowledge base can be used to anticipate novices’ mistakes and identify factors that would affect performance, such as high memory demand, attentional tunneling or distraction, time pressure and high cognitive workload, and poor situational awareness. For example, our results from the HDT show that the gaze transitions to primary variables are higher during success than in failure tasks. This can be used to provide automated feedback to novice operators to orient their gaze patterns. Providing such automated feedback is critical since the availability of expert instructors is decreasing, mainly due to retirement (Komulainen and Sannerud, 2018). In addition, experts’ gaze patterns can be generated with the proposed HDT and can be used to guide novice operators to orient their gaze patterns. There is evidence that guiding novices with experts’ gaze patterns can enhance their performance in tasks involving conflict detection performed by Air Traffic Controllers (Kang and Landry, 2014) and surgical tasks (Vine et al., 2013; Melnyk et al., 2021). Also, the HDT-based strategy can perform well in terms of the qualities that make up a reliable operator training assessment technique, such as consistency, repeatability, and neutrality.

Even after an operator learns the causal relationships in the process, it is possible that they can commit various errors during real-time plant operation due to inherent human frailties (Shahab et al., 2022a). Errors are the outcome of decision-based failures brought on by limits in human cognition, so they are particularly important when control room operators are dealing with abnormal situations. During such instances, many alarms from different parts of the process are triggered simultaneously, making it difficult for the operator to decide on the correct recovery strategy, especially within a short time frame (Naderpour et al., 2015). When confronted with complex situations, operators often find it challenging to judge which portion should be prioritized. This leads to an increase in their mental workload. The proposed human digital twin can help predict operators’ responses during novel abnormal situations and whether they would be able to handle such conditions during real plant operation. Additionally, temporal elements can be updated to keep track of the changing mental models of the human operator, such as how their behavior changes over time in response to process demands. It can thus be used to assist the operator by providing relevant cues and hence enable better situational awareness.

Even though our results indicated that the HDT performance is in general agreement with the performance of the human operators, the proposed HDT has certain limitations. For example, the current HDT is restricted to cases where only one abnormality occurs at a time. We intend to expand the model in the future to address multiple simultaneous abnormalities. In the future, we also plan to incorporate operator-specific differences in the HDT model and perform large-scale experiments. Further, the robustness of the HDT can also be evaluated using electroencephalography (EEG) data. For instance, Kim et al. (2019) developed an ACT-R with EEG simulation to estimate the operator’s mental workload during the air force multi-attribute task battery. The authors validated the model with the mental workload of the human operators obtained from EEG during the same task. Our previous studies with human participants reveal that EEG-based cognitive measures can be used to track the learning progress of operators (Iqbal et al., 2021). This can be used to evaluate the robustness of the human digital twin of the process operator.

## Data availability statement

The original contributions presented in this study are included in this article/supplementary material, further inquiries can be directed to the corresponding authors.

## Author contributions

All authors listed have made a substantial, direct, and intellectual contribution to the work, and approved it for publication.
